# The role of AMPKα2 in cardiomyocytes anoxia/reoxygenation injury mediated by Cl^-^

**DOI:** 10.1186/1479-5876-10-S2-A60

**Published:** 2012-10-17

**Authors:** Dan Liu, Sun Dian, Bin Zhong, Shu Zeng, Ming He

**Affiliations:** 1 Department of Pharmacology & Molecular Therapeutics, Nanchang University School of Pharmaceutical Science, Nanchang 330006, China

## Background

During anoxia/reoxygenation (A/R) injury, intracellular chloride ion concentration ([Cl^-^]_i_) homeostasis may play a role in maintaining the normal physiological function of cardiomyocytes. The cells protection was induced by Cl^-^-free when they were subjected to A/R injury, and we have found involvement of AMP-activated protein kinase-α2(AMPK-α2) in A/R injury. In the current study we investigated the mechanism of Cl^-^-free induced protection agaist A/R injury in H9c2 cells.

## Methods

AMPKα2 shRNA recombinant plasmid was contructed by using pSuper.Retro vector. The H9c2 cells were randomly divided into five groups: (1) Control group; (2) A/R group; (3) removal of extracellular Cl^-^+A/R group (Cl^-^-free A/R group); (4) pSuper+ Cl^-^-free A/R group; (5) pS-AMPKα2+ Cl^-^-free A/R group. The AMPKα2 protein expression was detected by western blotting. The activity of LDH was determined by auto-biochemistry analysator. Cells viability was analyzed by MTT, MDA, SOD and GSH-Px activity in H9c2 were detected by kits. The level of intracellular ROS, the percentage of apoptosis and the mitochondria membrane potential were measured by flow cytometry.

## Results

AMPKα2 shRNA recombinant plasmid was contructed successfully. A/R injury obviously decreased H9c2 cell viability, activity of SOD and GSH-Px. Cl^-^-free A/R group has been shown to produce a protective effect against A/R injury by increasing antioxidant enzyme. Once knockdowning the level of AMPKα2, the protective effect against A/R injury mediated by Cl^-^-subsitution apparently disappeared. Its cell viability, activities of SOD and GSH-Px, the mitochondria membrane potential were decreased while LDH activity and the level of ROS and apoptosis were remarkably increased in H9c2 cells compared with Cl^-^-free A/R group (*p*<0.01). There was no significant difference between Cl^-^-free A/R group and pSuper+ Cl^-^-free A/R group.

## Conclusion

AMPKα2 participated in the protective effect against A/R injury produced by administration with Cl^-^-free, and shAMPKα2 could abolish the protective effect. The mechanism underlying Cl^-^-free agaist A/R injury is mainly that of low [Cl^-^]_i_ attenuate oxidative stress by AMPKα2.

